# Triple Hydrophilic Statistical Terpolymers via RAFT Polymerization: Synthesis and Properties in Aqueous Solutions

**DOI:** 10.3390/polym15112492

**Published:** 2023-05-29

**Authors:** Dimitrios Vagenas, Stergios Pispas

**Affiliations:** Theoretical and Physical Chemistry Institute, National Hellenic Research Foundation, 48 Vassileos Constantinou Ave., 11635 Athens, Greece; dimitrisv98@gmail.com

**Keywords:** triple hydrophilic copolymers, responsive copolymers, RAFT polymerization, self-assembly, nanoaggregates

## Abstract

In this work, we report the synthesis of novel triple hydrophilic statistical terpolymers consisting of three different methacrylate monomers with varying degrees of responsivity to solution conditions. Terpolymers of the type poly(di(ethylene glycol) methyl ether methacrylate-co-2-(dimethylamino)ethylmethacrylate-co-oligoethylene glycol methyl ether methacrylate), P(DEGMA-co-DMAEMA-co-OEGMA), and of different compositions, were prepared by using the RAFT methodology. Their molecular characterization was carried out using size exclusion chromatography (SEC) and spectroscopic techniques, including ^1^H-NMR and ATR-FTIR. Studies in dilute aqueous media by dynamic and electrophoretic light scattering (DLS and ELS) show their potential responsiveness regarding changes in temperature, pH, and kosmotropic salt concentration. Finally, the change in hydrophilic/hydrophobic balance of the formed terpolymer nanoparticles during heating and cooling was studied using fluorescence spectroscopy (FS) in conjunction with pyrene giving additional information on the responsiveness and internal structure of the self-assembled nanoaggregates.

## 1. Introduction

Over the last few years, the fabrication of polymers, which demonstrate fascinating properties in solutions and can be applied in numerous studies, most of the time concerning the biomedical field, has been accelerated due to the tremendous evolution of polymerization techniques. Reversible addition fragmentation chain transfer (RAFT) is such a polymerization technique, providing versatility, control, and “livingness” to the synthesis process despite its radical attributes. Via this technique, it is possible to synthesize copolymers of different architectures and molecular weights, with a significantly low-molecular-mass dispersity and high end-group functionality [1]. Their induced self-assembly in aqueous solutions, regardless of their architecture, makes them ideal candidates for biomedical applications, such as drug delivery, gene delivery, or even tissue engineering [2,3]. In most cases, block copolymers are the epicenter of research, mainly because of their unique self-assembly in aqueous solutions, ending up in micelle formation, a well-defined morphology where nanoscale particles are concerned. However, the synthesis of block copolymers can be difficult and time-consuming due to the sequential addition of monomers for each block and sometimes the postpolymerization treatment that may be necessary. On the other hand, statistical (or random) copolymers can be prepared via a one-step reaction, applying the RAFT technique, given the potential to copolymerize two or more monomers simultaneously [4]. It is well known that the self-assembly of statistical copolymers provides less-well-defined nanostructures, as monomers/segments are randomly distributed along the polymer chains. However, supramolecular interactions occur in such systems, resulting in a variety of morphologies, which are worthy of further study. In fact, their nanostructures could be proven essential concerning the efficiency either of drug encapsulation or of its release. 

The stimuli responsiveness of polymers is also an issue that is attracting a lot of scientific interest nowadays. Specifically, it concerns polymers with monomeric units, which tend to respond to external stimuli, such as change in temperature and pH, or even to the presence of a salt. Influence from external stimuli is a factor that makes these copolymers “smart” and ideal for releasing drugs and other cargos under specific conditions of their surroundings. Multiple studies have proven that thermoresponsive polymers are subjected to a volume phase transition at a specific temperature range, resulting in a change in their solubility [5]. Such a transition is considered to be a direct consequence of the lowest critical solution temperature (LCST), which is caused by the incorporation of certain monomers to the final polymer chain [6,7]. Below LCST, polymer chains interact with water molecules via hydrogen bonding, retaining their solvophilicity, but when the temperature exceeds this critical point, hydrogen bonds break and polymer chains collapse resulting in the aggregation and dominance of hydrophobic interactions. Polymers that respond to pH changes, undergo similar structural and property changes as thermoresponsive polymers [8]. The fact that these two responses can be combined in a macromolecule raises great interest considering the study of the polymer self-assembly under varying environmental conditions. The presence of salt in a polymer solution is another factor of great significance as it provokes either the salting-in or the salting-out effect of the polymer chains, mainly depending on the polymer/salt nature and concentration [9]. When “pure” water is the solvent of the system, water molecules are located around polymer chains, which are associated through hydrogen bonds, creating hydrated layers. It is known from other studies that the hydrated layers are affected both by interactions between water and salt anions and polar groups of polymer with cations, dropping the LCST value [10]. 

At this point it is important to note the role of polymer amphiphilicity on its self-assembly behavior. It is well known that amphiphilic, random copolymers form nanoaggregates in aqueous solutions constituted of compact hydrophobic domains (cores), which are covered by the remaining hydrophilic segments. The balance between hydrophilic and hydrophobic segments is considered of great importance as far as the polymer self-assembly is concerned, especially when water is chosen as the solvent [11]. However, there are few reports regarding the self-assembly of hydrophilic, random copolymers, which exhibit conformational changes in response to external stimuli. 

So, it became essential for our group to focus on the self-assembly of hydrophilic, random copolymers, scrutinizing the resulting nanoparticle size and structure and investigating the influence of external stimuli on their characteristics, as well as that of copolymer composition. Our work is based on the synthesis of triple hydrophilic, random copolymers via RAFT polymerization, consisting of three different methacrylates: di(ethylene glycol) methyl ether methacrylate (DEGMA), 2-(dimethylamino)ethyl methacrylate (DMAEMA), and oligo(ethylene glycol) methacrylate (OEGMA_475_). Homopolymers of the aforementioned methacrylates are known both for their hydrophilicity at room temperature and stimuli responsive properties. In particular, PDEGMA has a LCST value at 27 °C, PDMAEMA at the temperature range of 45–60 °C and POEGMA at a much broader range of 25–90 °C depending on the length of the ethylene glycol moieties [12,13,14]. Indeed, PDMAEMA is well known for its pH responsiveness due to the existence of a tertiary amino group on its side chain. Theoretically, it is expected that the system’s hydrophobic/hydrophilic balance will shift toward hydrophobic mainly because of the DEGMA and DMAEMA segments present, which respond either to temperature or pH changes. Of course, one should also take into account the terpolymer content in OEGMA, a factor that may also determine its self-assembly behavior [11,15]. Finally, terpolymer solutions with two different concentrations of the kosmotropic salt Na_2_SO_4_ were investigated in order to explore the salting-out effects on the terpolymer behavior. 

## 2. Materials and Methods

### 2.1. Materials

Monomers, di(ethylene glycol) methyl ether methacrylate (DEGMA), 2-(dimethylamino)ethyl methacrylate (DMAEMA), and oligo(ethylene glycol) methacrylate (OEGMA) (average M_n_ = 475 g/mol) were purified, passing through a column packed with inhibitor removing resins 311,340 and 311,332 from Sigma-Aldrich (Athens, Greece). 2,2-azobis(isobutyronitrile) (AIBN) was recrystallized from methanol and was used as the radical initiator. 1,4-dioxane (99.8% pure) was chosen as the solvent of the reaction and was dried using molecular sieves. 4-cyano-4-(phenylcarbonothioylthio) pentanoic acid (CPAD) was the chain transfer agent of the reaction, and n-hexane, tetrahydrofuran (THF), sodium sulfate (Na_2_SO_4_), pyrene, and deuterated chloroform (CDCl_3_) were used as received (all from Sigma-Aldrich).

### 2.2. Synthesis of P(DEGMA-co-DMAEMA-co-OEGMA) Terpolymers

The synthesis of three linear, random P(DEGMA-co-DMAEMA-co-OEGMA) terpolymers with a different monomer composition in each case, was achieved via one-step RAFT polymerization. Purified monomers, AIBN and CPAD, were placed in a 25 mL round-bottom flask and were all dissolved in 1,4-dioxane. The CPAD:AIBN ratio was adjusted at 2:1 (mol), and the targeted polymer molecular weight was 20,000 g/mol. After the homogenization of the mixture under stirring, the flask was sealed with a rubber septum, and deoxygenation of the polymerization solution was achieved via nitrogen bubbling for 20 min. Then, the flask was put in oil bath at 70 °C while stirring and left there for 24 h. Afterward, the flask was put directly at −20 °C for 20 min and finally exposed to air, terminating the polymerization. Then, the terpolymers were precipitated in hexane excess, and the final products were collected and placed in a vacuum oven for 48 h to dry. 

### 2.3. Self-Assembly of P(DEGMA-co-DMAEMA-co-OEGMA) in Aqueous Media

The three hydrophilic terpolymers were studied considering their ability to self-assemble in aqueous solutions following a relatively simple protocol. Deionized water was selected as the solvent as all three terpolymers were diluted directly in it at pH = 7. In each case, 10 mL aqueous solutions were prepared with stable terpolymer concentration regulated at 1 × 10^−3^ g/mL and were left for approximately 24 h in order to be equilibrated. Due to DMAEMA’s pH-responsive character, terpolymer solution pH was regulated at pHs = 3 and 10 by adding the appropriate amount of HCl 0.1 M and NaOH 0.1 M, respectively. Concerning dynamic light-scattering studies, all solutions were filtered through hydrophilic PVDF 0.45 μm disposable filters before measurements.

### 2.4. Salt Effects

The coexistence of a salt, such as Na_2_SO_4_ and terpolymer, is a case worth investigating due to the possible conformation/aggregation changes in the polymer self-assembled nanostructures. These studies were conducted only on proven thermoresponsive terpolymers to investigate the influence of the induced salting-out effect on these macromolecules. Solutions were prepared in two different salt concentrations, 0.15 M and 0.3 M, respectively, where terpolymer concentration remained constant (at 1 × 10^−3^ g/mL), and afterward, part of them was measured via dynamic light scattering to ascertain if the salt concentration amplifies the salting-out effect. 

### 2.5. Characterization Methods

#### 2.5.1. Size Exclusion Chromatography (SEC)

**Figure 1:**
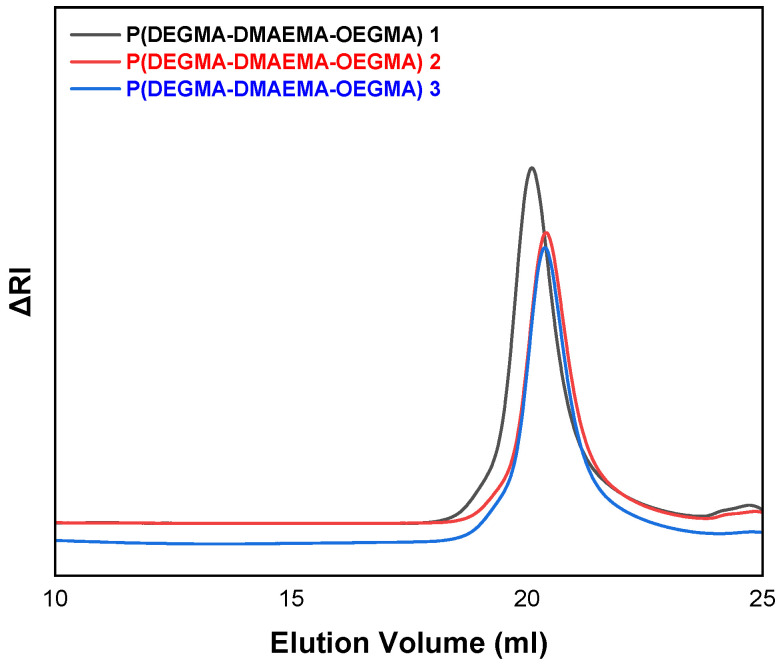
SEC chromatograms for terpolymers P-1, P-2, and P-3.

The molecular mass and the mass distributions of the synthesized terpolymers were determined by size exclusion chromatography. A Waters SEC set-up was used, consisting of an isocratic Waters 1515 pump, a set of three µ-Styragel mixed-composition separation columns (10^2^–10^6^ Å pore range), a Waters 2414 refractive index detector (at 40 °C), and Breeze software for SEC set-up control. Tetrahydrofuran was used as the solvent, which contained 5% *v/v* triethylamine, at a flow rate of 1 mL/min at 30 °C. The chromatograms of the obtained terpolymers are shown in Figure 1.

#### 2.5.2. Proton Nuclear Magnetic Resonance Spectroscopy (^1^H-NMR)

^1^H NMR measurements of terpolymer samples were performed on a Varian 300 (300 MHz) spectrometer using the Vjnmr Software for spectra acquisition. CDCl_3_ was used as the solvent for sample preparation (c ≈ 14 mg/mL). Chemical shifts are given in parts per million (ppm) using tetramethylsilane as the internal reference, and the results were analyzed by MestReNova Software (version 6.0.2-5475) from MestReLabs (Santiago de Compostela, Spain).

#### 2.5.3. Attenuated Total Reflectance-Fourier Transform Infrared (ATR–FTIR) Spectroscopy

FTIR spectra of dry solid terpolymer samples were recorded on a Bruker (Billerica, MA, USA) Equinox 55 Fourier transform spectrometer, equipped with a single-bounce ATR diamond accessory (Dura-Samp1IR II by SensIR Technologies, Danbury, CT, USA). Every spectrum was received as the average of 64 scans collected in the 5000 to 500 cm^−1^ spectral range and at 4 cm^−1^ resolution.

#### 2.5.4. Dynamic Light Scattering (DLS)

DLS studies were carried out with an ALV/GS-3 compact goniometer system (ALV GmbH, Hessen, Germany) with a JDS Uniphase 22 mW He–Ne laser, operating at 632.8 nm wavelength. The system is equipped with an ALV/LSE-5003 light-scattering electronics unit used for stepper motor drive and limit switch control and an ALV-5000/EPP multi-τ correlator including 288 channels. The obtained autocorrelation functions (and the simultaneously recorded light-scattering intensity) were the average of five measurements at a goniometer angle of 90° and were analyzed by the cumulants method and the CONTIN algorithm. All aqueous solutions were filtered through 0.45 µm hydrophilic PVDF filters before measurements.

#### 2.5.5. Electrophoretic Light Scattering (ELS)

Zeta potential values, which are directly related to the surface charge of polymer particles, were measured by electrophoretic light-scattering experiments conducted on a Nano Zeta Sizer instrument from Malvern, which is equipped with a 4 mW He–Ne, operating at 633 nm and a scattering angle of 173°. Each measurement was the average of approximately 20 repeated scans, and the obtained data were analyzed by the Smoluchowski equation.

#### 2.5.6. Fluorescence Spectroscopy (FS)

The pyrene fluorescence spectra in the presence of terpolymers and under different solution conditions were recorded with a NanoLog Fluorimeter (Horiba Jobin Yvon, Kyoto, Japan), using a laser diode as the excitation source (NanoLED, 440 nm, pulse width 100 ps) and a UV TBX-PMT series detector (250–850 nm) by Horiba Jobin Yvon. Solutions of each terpolymer were prepared at constant polymer concentration. Pyrene solution in acetone at a ratio of 1 µL/mL was added to each vial. The samples were kept at rest for 24 h to ensure encapsulation of pyrene into the hydrophobic domains of the polymer aggregates and evaporation of acetone. The excitation wavelength used for the measurements was 335 nm. Emission spectra were recorded in the spectral range of 355–640 nm. The ratio I_1_/I_3_, i.e., the ratio of intensities of the first and third vibronic peaks in pyrene fluorescence spectra, was utilized in order to access the hydrophobicity of pyrene environment.

## 3. Results and Discussion

### 3.1. Synthesis and Molecular Characterization of the Terpolymers

**Scheme 1:**
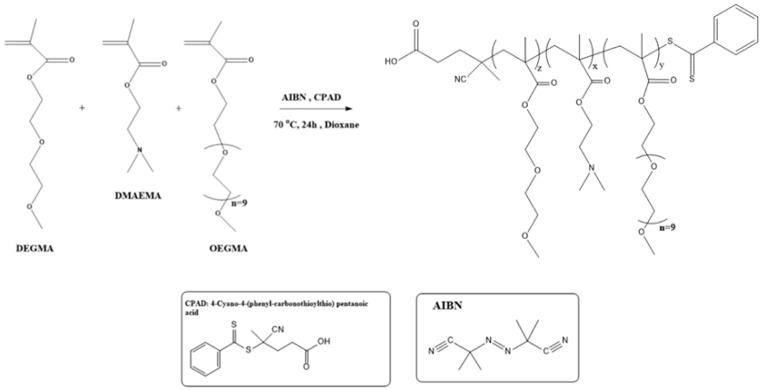
Synthetic route for obtaining P(DEGMA-co-DMAEMA-co-OEGMA) terpolymers via RAFT polymerization.

The synthesis of three P(DEGMA-co-DMAEMA-co-OEGMA) statistical terpolymers was conducted via RAFT polymerization as shown in Scheme 1. CPAD was chosen to be the chain transfer agent, as its utility to the polymerization of a broad spectrum of methacrylates is also proven in other works [11,16]. The three terpolymers have different monomer compositions by weight, but the targeted molecular weight was the same for all of them. Size exclusion chromatography (SEC) was the first characterization method performed to inspect if the synthesis of the three terpolymers was successful, relying on the control of their molecular weights and their distribution values. All three chromatograms are shown in Figure 1, composed of unimodal peaks indicating the controlled simultaneous polymerization of the three monomers in all cases, resulting molecular weights close to the stoichiometric ones, and low-molecular-mass distribution values consistent with the theoretical background of RAFT polymerization. The incorporation of monomers into the terpolymer chains may be assumed to have been achieved statistically, as these monomers were copolymerized in pairs randomly in other studies [17,18]. Regardless of the controlled and “living” polymerization nature, the careful setting of the reaction concerning the regulation of the appropriate conditions as well as the selection of the appropriate initiator and solvent, is considered to play a vital role. The molecular characteristics of the three terpolymers obtained by SEC are showcased in Table 1. 

**Figure 2:**
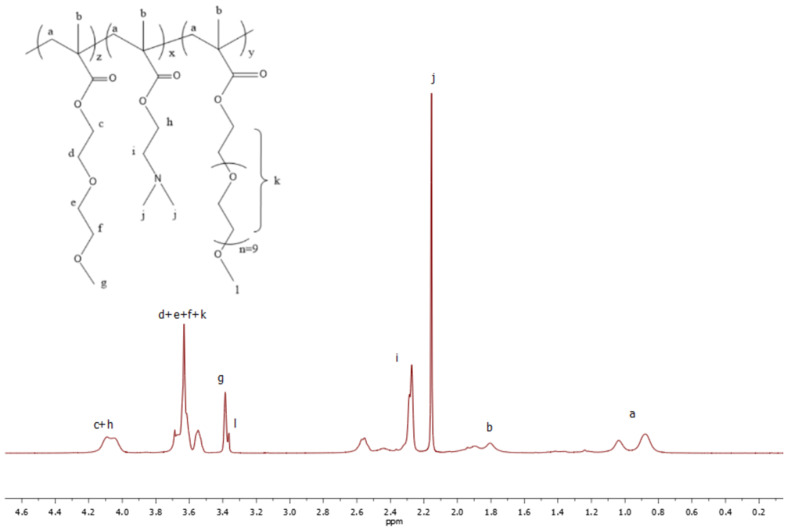
^1^H-NMR spectra of P-3 P(DEGMA-co-DMAEMA-co-OEGMA) terpolymers. Letters in the spectrum correspond to the protons shown in the terpolymer chemical structure on the upper left part of the figure.

**Figure 3:**
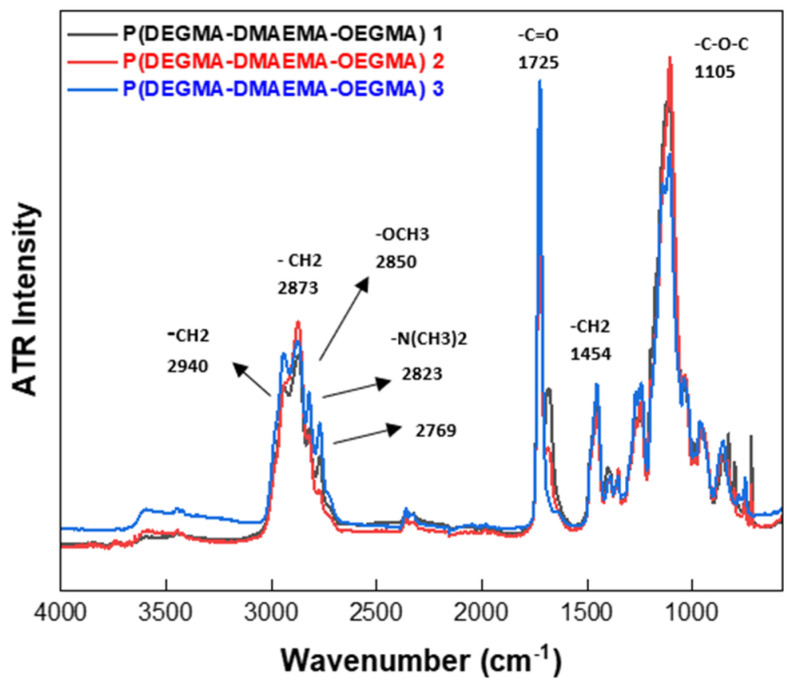
ATR−FTIR spectra of P(DEGMA-co-DMAEMA-co-OEGMA) terpolymers.

As for the identification of the chemical structure and composition of the terpolymers, ^1^H-NMR (Figure 2) and FTIR (Figure 3) experiments were carried out. The first technique verified the incorporation of all three monomers in the terpolymer chain qualitatively and quantitatively, and the second one provided qualitative results for each monomer content. Concerning the ^1^H-NMR spectra (Figure 2, and Figures S1–S3 and Tables S1 and S2 in Supplementary Materials), it is obvious that their quantitative analysis becomes extremely difficult due to the multiple overlaps that occur due to the similar chemical environment of protons along the polymer structure. Nevertheless, a quantitative analysis was achieved due to the differentiation of the methoxy protons (-OCH_3_) of DEGMA and OEGMA, at 3.38 and 3.36 ppm, respectively [19]. The peak at 2.16 ppm, which belongs to the tertiary amino group protons of DMAEMA, was used as the reference peak [20]. Indicatively, the ^1^H-NMR spectrum of P-3 terpolymer is presented in Figure 2. As for P-2 terpolymer, the methoxy protons of DEGMA and OEGMA could not be distinguished and showed low signals, factors that prevented a quantitative analysis of the copolymer composition. More details can be found in the Supplementary Materials Section (Figures S1–S3 and Tables S1 and S2). The ATR-FTIR spectra of the three terpolymers are presented in Figure 3.

### 3.2. Self-Assembly in Aqueous Media

**Figure 4:**
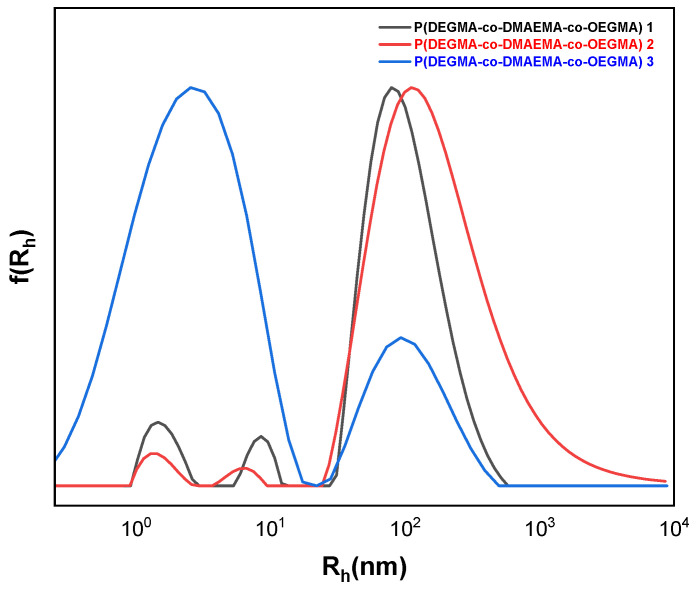
Size distributions from CONTIN for the three terpolymers at 25 °C and pH = 7.

Aqueous solutions of the three terpolymers were prepared via the direct dilution in deionized water as described in the experimental section. The nanoscale self-assembly of the three terpolymers was studied via dynamic light-scattering (DLS) measurements, determining parameters, such as the apparent hydrodynamic radius (R_h_), the scattered light intensity, and the size polydispersity index (PDI), of the formed nanoaggregates. In the early stages of the experiments, measurements were conducted at 25 °C and at pH = 7 for all polymer solutions, and their size distributions are shown in Figure 4. 

It is obvious that the terpolymers tend to self-organize via the formation of nanostructures of varying size, in a manner related to the composition of each terpolymer. Scattered light intensity values indicate the mass of the formed nanoparticles. In this case, the values fluctuate from 30 to 90 kHz, indicating the existence of relatively small nanoparticles in the solutions. However, concerning the P-1 and P-2 terpolymers, in Figure 4, three nanoparticle populations are presented, with the most dominant one approaching R_h_ values of 100 nm, a representation though that does not fit completely with the relatively low scattered intensity values. Such size distribution curves are indicative of the existence of large nanoaggregates (globular structures), most probably swelled with solvent. It is known that the f(R_h_) curves are strongly influenced by scattered intensity arising from the different species in solution, which tends to increase proportionally with the nanoparticle size [21]. Therefore, the size distribution of the P-1 and P-2 terpolymers provides information concerning the existence of both single-chain and multimolecular structures, but the intensity values do not necessarily indicate the dominance of the large aggregates in terms of weight fraction at 25 °C. P-3 terpolymer size distribution represents a broad range of particles of relatively small R_h_ values (1–20 nm) as the dominant species, and this is in agreement with the low intensity values determined. In this case, it is safer to assume that small, including single-chain species and low aggregation number aggregates, are the dominant populations in solution. This differentiation with the other two terpolymers may result from the lower OEGMA content of P-3 and will be discussed further. Based on the aforementioned, all three terpolymers self-assemble mostly intramolecularly in their single-chain form in aqueous solutions. In fact, these results are in agreement with other studies where it was proven that the self-assembly of statistical copolymers of methacrylates is based on the random distribution of free polymer chains and then their induced random coil formation [19]. As for globular structures, it is known that the hydrophobic part of the polymer folds in and forms a packed nucleus and the hydrophilic part distributes around it [20]. Nevertheless, in this study, the synthesized copolymers are hydrophilic at room temperature and have a neutral pH, raising questions of the existence of aggregates of the core–shell-type. The random distribution of monomers along the polymer chain is considered to provoke Van der Walls and dipole–dipole interactions as well as interactions between solvent molecules and side chains, inducing the formation of hydrophilic nanoaggregates with no hydrophobic cores [22,23].

As mentioned previously, P-3 terpolymer self-assembly progresses intensively through single-polymer chains rather than through multimolecular nanoaggregates, in comparison to the other two terpolymers showing a broader size distributions. This differentiation could be attributed to the composition of P-3, which contains 13% OEGMA per weight. Both the low content of OEGMA units and the excess of DEGMA and DMAEMA units may induce the intramolecular aggregation of polymer chains, which afterward collapse and result in unimers with random coil formation and rather globular structures [24,25].

### 3.3. Thermoresponsiveness of P(DEGMA-co-DMAEMA-co-OEGMA) Terpolymers

The three synthesized terpolymers are expected to respond with a temperature change due to the thermoresponsive nature of the monomers that are constituted. Notably, the homopolymers of DEGMA, DMAEMA, and OEGMA show LCST behavior, which strongly depends on the hydrophobic/hydrophilic balance when it comes to statistical copolymers. At this point, it is important to provide a molecular background concerning the intermolecular interactions happening in such aqueous solutions. Under the lower critical solution temperature, aqueous phase predominates due to the multiple hydrogen bonding of polymer functional groups with water molecules. Specifically, ether oxygens of ethylene glycol groups and ester groups form hydrogen bonds with water molecules relatively easily. Tertiary nitrogen of DMAEMA also forms hydrogen bonds but less effectively, due to its overall interception from the two hydrophobic methyl groups and the rest side chain. The DEGMA part is close to its LCST at 25 °C. When PDMAEMA LCST is overcome, hydrogen bonds break, and, as a result, polymer chains approach each other favoring hydrophobic interactions and aggregate. Furthermore, it has been found that a copolymer’s LCST can shift upward or downward depending on its OEGMA content [26]. This monomer has been proven to actively impact a copolymer’s self-assembly in aqueous media, resulting in stabilized structures, and, due to its biocompatibility, it is of interest for biological applications. Moreover, its intense hydrophilicity tends to increase the LCST of copolymer systems of which it is a part, meaning that with its use, it becomes possible to tune the thermoresponsiveness of a copolymer. 

**Figure 5:**
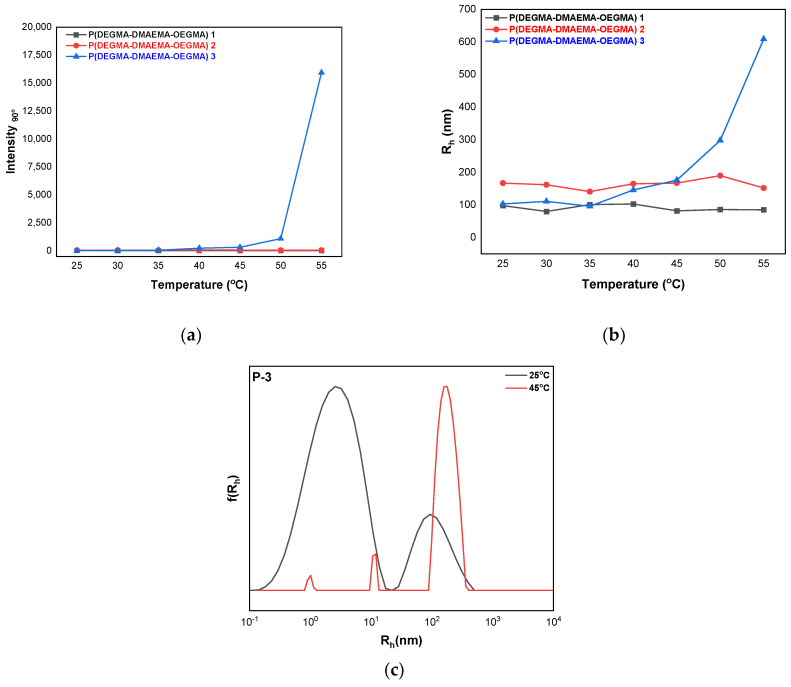
(**a**) Scattered light intensity as a function of temperature for the three terpolymers at pH = 7. (**b**) Hydrodynamic radius as a function of temperature for the three terpolymers at pH = 7. (**c**) Size distribution of terpolymer P-3 at 25 and 45 °C at pH = 7.

Dynamic light-scattering measurements were carried out again for the three polymeric aqueous solutions but this time at a temperature range starting from 25 °C and ending at 55 °C, emphasizing the analysis of the resulting values of scattered light intensity, and R_h_ and PDI correlated with the temperature increase in each case. From the three terpolymers, only the third one showed significant thermoresponsiveness as presented in Figure 5. 

Terpolymers P-1 and P-2 show notable structural stability with temperature increases, a fact that is justified by the relative stable values of the scattered light intensity and hydrodynamic radius, meaning that neither a further aggregation nor a conformational changes take place. This finding can be attributed to the OEGMA content of the terpolymers, as in both cases, it is kept at high levels. As for terpolymer P-3, the regulation of the OEGMA content at 13% per weight seems to favor thermoresponsiveness, a fact that is presented well in Figure 5a,b, as scattered light intensity shows a slight increase after 35 °C and then drastically increases up to 55 °C. This rapid increase in intensity value indicates the aggregation of free polymer chains forming mesoglobules, which is also supported by the increase in R_h_ values. Therefore, the LCST of P-3 was found to be at 35 °C. The size distribution also depicts the shift to higher R_h_ values and the formation of nanoaggregates for terpolymer P-3 with temperature increases (Figure 5c). At this point, it must be noted that at 55 °C, the aggregation was so intense that a multiscattering effect was observed by the naked eye. A phenomenon such as this can be attributed to the hydrophobic character of polymer chains at that temperature, as the hydrophobic/hydrophilic balance is shifted toward hydrophobic, repelling water molecules out of the polymer coils and creating larger and more compact multimolecular structures. 

**Figure 6:**
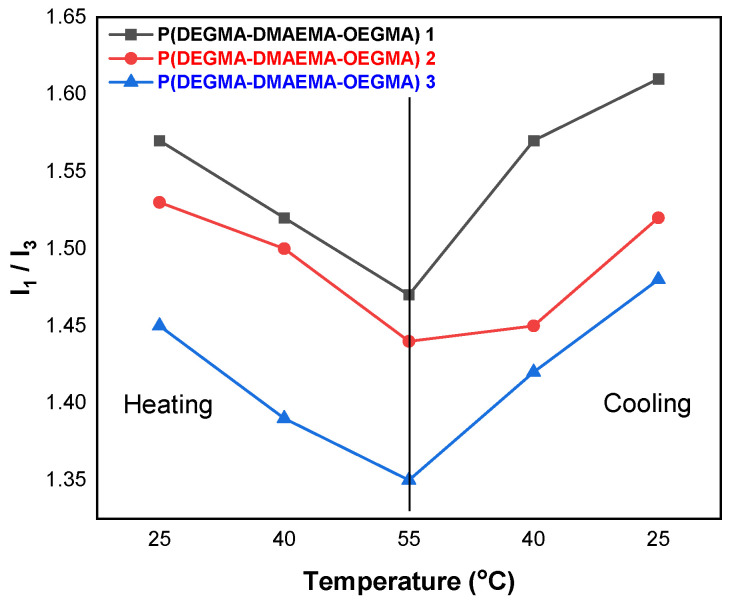
I_1_/I_3_ ratio of pyrene probe in terpolymer aqueous solutions as a function of temperature.

The above observations can be also supported by fluorescence spectroscopy measurements of the same solutions. Terpolymers do not fluoresce; thus, pyrene, which is a fluorescent probe, was added to the solutions. Pyrene is known for its characteristic emission spectra from which significant information concerning the microenvironment polarity around the probe can be extracted [27]. The I_1_/I_3_ pyrene ratio is characteristic for the environmental polarity at the molecular level. Large values of this ratio are indicative of hydrophilic systems, and small ones are indicative of hydrophobic systems. It becomes apparent that pyrene fluorescence measurements should be conducted to ensure the balance shift from hydrophilic to hydrophobic within the terpolymer nanoassemblies upon heating and the reverse effect upon cooling, as it is presented in Figure 6.

It is worth noting that all three terpolymers shift their hydrophilic/hydrophobic balance toward the hydrophobic side upon heating, with the third terpolymer showing the largest shift, a fact that can be correlated with its significant thermoresponsiveness due to the specific chemical composition. In Figure 6, it seems that all three terpolymers show temperature-induced changes, a finding that can be supported at the molecular level, as pyrene fluorescence depends on environment micropolarity changes. It is important to point out that the LCST realization of P-3 terpolymer cannot be justified via fluorescence measurements, as it is considered a nanoscale phenomenon, which depicts the aggregation of nanodomains between polymer chains. However, fluorescence measurements illustrate the temperature-induced modifications for the three terpolymers at a molecular level, shifting the hydrophilic–hydrophobic balance. Another interesting finding is that all three terpolymers seem to return to their previous (hydrophilic) state upon cooling from 55 °C to 25 °C; thus, these temperature-induced structural changes in the terpolymers in water can be considered reversible.

### 3.4. pH Responsiveness of P(DEGMA-co-DMAEMA-co-OEGMA) Terpolymers

The existence of the DMAEMA units in all three terpolymers motivated studies on the potential response of the terpolymers following a change to a more acidic or basic solution pH. The DMAEMA monomer carries a tertiary amino group, which is partially protonated at a neutral pH, establishing a relative hydrophilic character along the polymer chain. At acidic pH values (pH~3), the amino group becomes fully protonated, intensifying the hydrophilicity and imparting polyelectrolyte properties [28]. In contrast, at basic pH values (pH~10), the amino group is deprotonated forcing the monomeric units to adapt hydrophobic characteristics, not favoring the formation of hydrogen bonds between water molecules and polymer chains [29]. 

Thus, it was decided to investigate the self-assembly of the three terpolymer solutions under basic conditions (pH = 10) at room temperature in order to examine the overall effect of the deprotonation of the DMAEMA tertiary amino group. The results of dynamic light-scattering measurements under these conditions are summarized in Table 2, also incorporating the results at a neutral pH for comparison.

The increase in light-scattering intensity for terpolymers P-1 and P-3 where the DMAEMA content is 36% and 44% per weight, respectively, is obvious and leads to the conclusion that the pH response of the terpolymer is strongly dependent on its composition. Terpolymer P-2, which possibly contains half the amount of DMAEMA (20%), does not respond to the pH change, retaining similar intensity values. This effect can be ascribed to the low DMAEMA content, resulting in less-exposed tertiary amino groups and thus no response to pH.

Thereafter, electrophoretic light-scattering measurements were carried out for all three terpolymers at room temperature and at three different pH values to evaluate any possible change in the surface charge of the macromolecules/aggregates in solution. ζ-potential values for the three terpolymers at their respective pH are summarized in Table 3. After the adaptation of acidic conditions (pH~3) in the terpolymer solutions, positive superficial charge dominates along the polymer chains. This effect is more intense for the third terpolymer case where the OEGMA content is the lowest (13%) resulting in a more localized excess of tertiary amino groups. Under basic conditions, all three terpolymer solutions present negative ζ-potential values as expected. 

**Figure 7:**
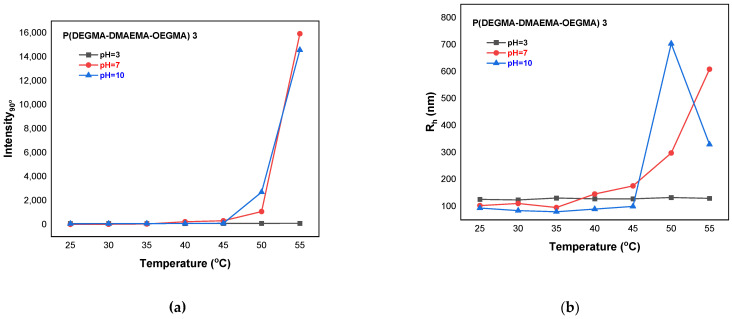
(**a**) Scattered light intensity as a function of temperature for terpolymer P-3 at three pH values. (**b**) Hydrodynamic radius as a function of temperature for terpolymer P-3 at three pH values.

The next step concerning the pH-responsiveness experiments was the investigation of the only thermoresponsive terpolymer (P-3), exposing it to both temperature and pH stimuli. To this end, dynamic light-scattering measurements were conducted for P-3 solutions at three different pH values (3, 7, and 10) and at the temperature range of 25–55 °C to inspect any potential structural or conformational changes. The light-scattering intensity and hydrodynamic radius as a function of temperature for each pH solution of terpolymer 3 are presented in Figure 7 showing some interesting trends. The Terpolymer P-3 at an acidic pH seems to lose its thermoresponsiveness as both the light-scattering intensity and the hydrodynamic radius remain the same from 25 to 55 °C. At a pH = 3, the DMAEMA units converted to extremely hydrophilic moieties and may influence the manifestation of the thermal response. As for the basic solution, thermoresponsiveness occurs but with a slight delay, with LCST being observed at 45 °C. Concerning the delay effect, it can be assumed that the DMAEMA units obtained a saturated hydrophobic character due to the basic pH of the solution and do not respond intensively to a temperature increase. In addition, a transition of the R_h_ values of the basic solution is observed at 55 °C. 

**Figure 8:**
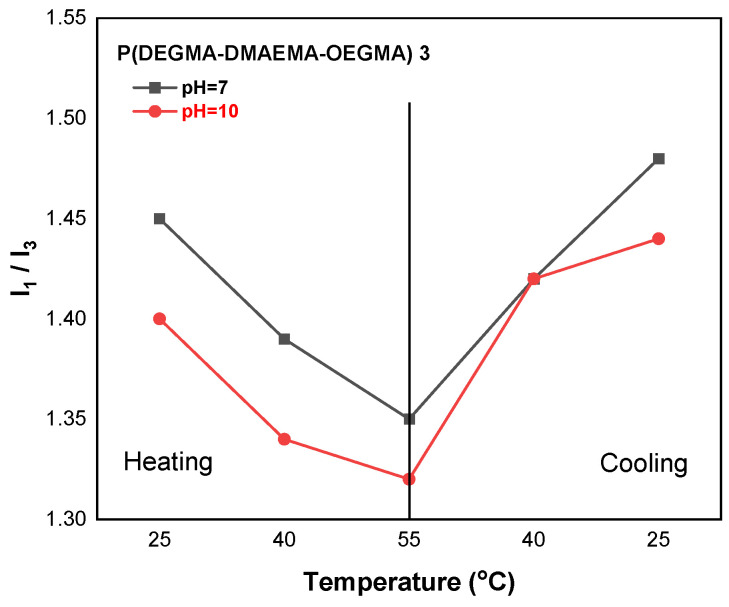
I_1_/I_3_ ratio of terpolymer P-3 aqueous solutions at pHs 7 and 10 as a function of temperature.

The transition of the hydrophilic to hydrophobic character of terpolymer P-3 due to the pH change is also proven through fluorescence experiments, probing changes in the micropolarity of the pyrene environment. As it is presented in Figure 8, terpolymer P-3 becomes more hydrophobic at a pH = 10 at room temperature but does not seem to undergo any further significant structural change concerning the solvophilicity balance. At a pH = 7, the basic terpolymer solution also returns to its previous state upon cooling, showing strong reversibility of the observed structural transition.

### 3.5. Salt Solutions of P(DEGMA-co-DMAEMA-co-OEGMA) Terpolymers

After studying the response of the terpolymers to external stimuli, we considered investigating the possible influence of Na_2_SO_4_ salt on the conformation/structure of the highly thermoresponsive P-3 terpolymer. Na_2_SO_4_ is known as a kosmotropic salt of the Hoffmeister series. Its anions tend to attract water molecules and hydrate, causing the corresponding dehydration of polymer chains, when they coexist in an aqueous solution. As a result, SO_4_^2−^ anions polarize the hydrogen bonds between water molecules and polymer chains, forcing the overexpose of hydrophobic parts of the polymer and inducing the so-called salting-out effect. Simultaneously, ethylene glycol groups are associated electrostatically with sodium cations, resulting in the partial destruction of the hydrated layer [10]. This effect enhances the aggregation between polymer chains, as hydrophobic interactions are favored, and decreases their solubility significantly. This means that the presence of a kosmotropic salt in a polymer solution has a direct influence on its LCST value [9,30] and therefore promotes the aggregation of polymer chains. 

**Figure 9:**
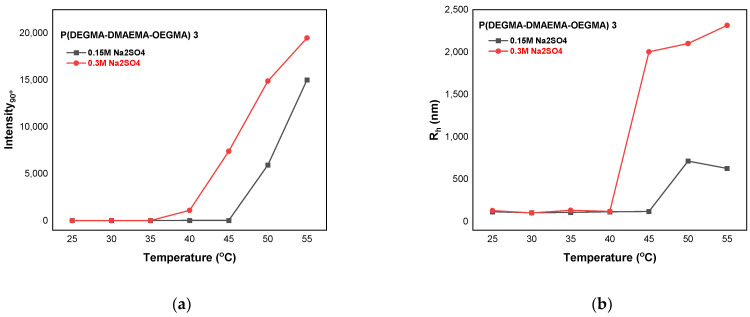
(**a**) Scattered intensity as a function of temperature for two terpolymer P-3 solutions with Na_2_SO_4_ concentrations at 0.15 and 0.3 M. (**b**) Hydrodynamic radius as a function of temperature for the same terpolymer solutions (c_pol_ = 1 × 10^−3^ g/mL).

Two solutions of Na_2_SO_4_ and terpolymer 3 were prepared in two different concentrations of salt at 0.15 and 0.3 M, maintaining a constant terpolymer concentration at 1 × 10^−3^ g/mL. These two salt concentrations were chosen to inspect the influence of salt concentration on both the terpolymer self-assembly and its thermoresponsiveness in water. To better understand this influence, dynamic light-scattering measurements were conducted, evaluating the two basic parameters: scattered light intensity and hydrodynamic radius, the values of which were correlated with temperature increases and are presented in Figure 9.

From the above results, some trends are observed concerning the influence of the salt concentration on the aggregation state of the terpolymer in solution. In the intensity plot, the concentration of the salt has an active role concerning the response of the macromolecule with a temperature increase. The more anions in the solution, the smaller the LCST value, determined as the temperature at the upturn of intensity. Apart from this, it is worth noticing that in the 0.3 M Na_2_SO_4_ solution, the intensity attains higher values in comparison to the 0.15 M solution, meaning that the aggregation of polymer chains is much more intense in the 0.3 M solution. The same trend is also depicted in the R_h_ transition with temperature increases, as the aggregates of the 0.3 M Na_2_SO_4_ solution show large size values. 

**Figure 10:**
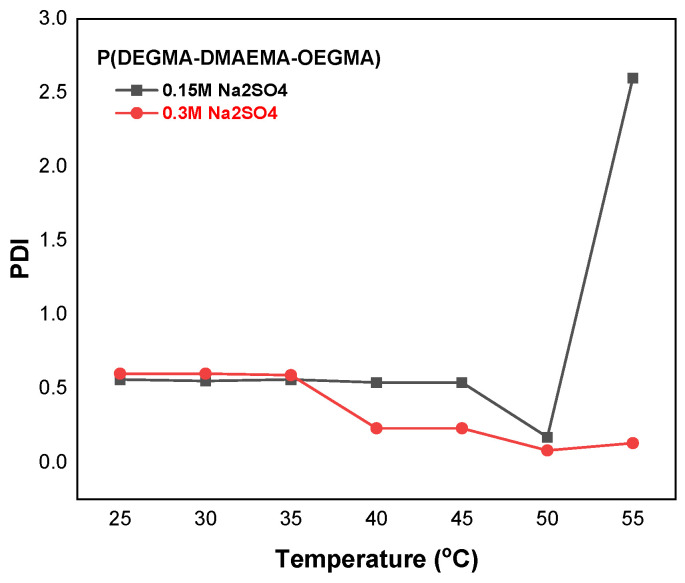
PDI from DLS as a function of temperature for terpolymer P-3 salt solutions at 0.15 and 0.3 M Na_2_SO_4_.

Furthermore, in the case of the coexistence of kosmotropic anions with polymer chains, significant changes concerning the polydispersity of the terpolymer are observed with temperature increases. In both salt concentrations, the PDI seems to decrease from the LCST and above and shows great dependance on the concentration. The 0.3 M Na_2_SO_4_ salt–polymer solution develops homogeneous and large nanoaggregates with the increase in temperature as its PDI reaches even values close to 0.1. The trend of the PDI’s transition with the increase in temperature for both salt–terpolymer solutions is presented in Figure 10.

## 4. Conclusions

Three statistical terpolymers of the type P(DEGMA-co-DMAEMA-co-OEGMA) were synthesized successfully via RAFT polymerization with different monomer compositions and were characterized molecularly. Self-assembly studies through methods, such as DLS, ELS, and FS, demonstrated that one out of the terpolymers, P-3, was highly thermoresponsive mainly because of its low OEGMA content. The terpolymer was found to form mainly single-chain globules at 25 °C. The terpolymers P-1 and P-3 with a relatively high DMAEMA content became more hydrophobic under basic conditions due to the deprotonation of the tertiary amino groups. The concentration of the kosmotropic salt Na_2_SO_4_ was proven to be a major influence on the self-assembly of terpolymer P-3 at the temperature range of 25–55 °C, resulting in larger aggregates in mass and size and lower size dispersity with temperature increases, thus verifying its salting-out effect on the terpolymer. The results presented contribute new knowledge to the rather scarce studies of multiresponsive statistical/random terpolymers, which can be potentially utilized as nanocarriers for bioactive compounds and used as simple models for protein aggregation and denaturation behavior in aqueous solutions.

## Figures and Tables

**Table 1:** Molecular characteristics of the P(DEGMA-co-DMAEMA-co-OEGMA) terpolymers.

Sample	M_w_ (×10^4^) (g/mol) ^a^	M_w_/M_n_^a^	Initial Feed Ratio ^d^ (DEGMA/DMAEMA/OEGMA)	%wt Compositon ^b,d^ (DEGMA/DMAEMA/OEGMA)	Molar Composition ^b,d^ (DEGMA/DMAEMA/OEGMA)
P-1	2.30	1.34	[0.44:1:0.34]	[12:36:52]	[0.28:1:0.48]
P-2	1.90	1.24	[1:0.59:0.38]	[40:20:40] ^c^	[1:0.59:0.38] ^c^
P-3	1.95	1.25	[0.88:1:0.17]	[43:44:13]	[0.82:1:0.1]

^a^ Determined by SEC. ^b^ Determined by ^1^H-NMR. ^c^ Theoretical. ^d^ [DEGMA:DMAEMA:OEGMA].

**Table 2:** DLS results of the three terpolymers at pHs 7 and 10 at 25 °C.

Sample	pH	I_90°_ (kHz)	R_h_ (nm)
P-1	7	39	98
	10	140	81
P-2	7	70	167
	10	67	92
P-3	7	23	2
	10	80	94

**Table 3:** ζ-potential values for the three terpolymer aqueous solutions at pHs 3, 7, and 10 at 25 °C.

Samplel	pH	ζ-Potential (mV)
P-1	3	+3.5
	7	+2
	10	−1.5
P-2	3	+5
	7	+0.7
	10	−1
P-3	3	+40
	7	−0.42
	10	−0.5

## Data Availability

Data are available on request.

## Supplementary Materials

The following supporting information can be downloaded at: https://www.mdpi.com/article/10.3390/polym15112492/s1. Figure S1. (a) ^1^H-NMR of P-1 terpolymer (b) line fitting of methoxy protons peak; Figure S2. (a) ^1^H-NMR of P-2 terpolymer (b) line fitting of methoxy protons peak.; Figure S3. (a) ^1^H-NMR of P-3 terpolymer (b) line fitting of methoxy protons peak; Table S1. Line fitting data for methoxy protons of DEGMA and OEGMA of terpolymer P-1; Table S2. Line fitting data for methoxy protons of DEGMA and OEGMA of terpolymer P-3.
