# Supplementary material for: Triple Hydrophilic Statistical Terpolymers via RAFT Polymerization: Synthesis and Properties in Aqueous Solutions
Source: Polymers (Basel). 2023 May 29;15(11):2492. doi: 10.3390/polym15112492 (PMC10255189; doi:10.3390/polym15112492)
Supplement: Supplementary file 1 [file polymers-15-02492-s001.zip › polymers-2398985-supplementary.pdf]

Supplementary Materials

Triple Hydrophilic Statistical Terpolymers via RAFT Polymerization: Synthesis and Properties in Aqueous Solutions

Dimitrios Vagenas [1], and Stergios Pispas [1,*]

[1] Theoretical and Physical Chemistry Institute, National Hellenic Research Foundation, 48 Vassileos Constantinou Ave., 11635 Athens, Greece; dimitrisv98@gmail.com; pispas@eie.gr

* Correspondence: pispas@eie.gr;

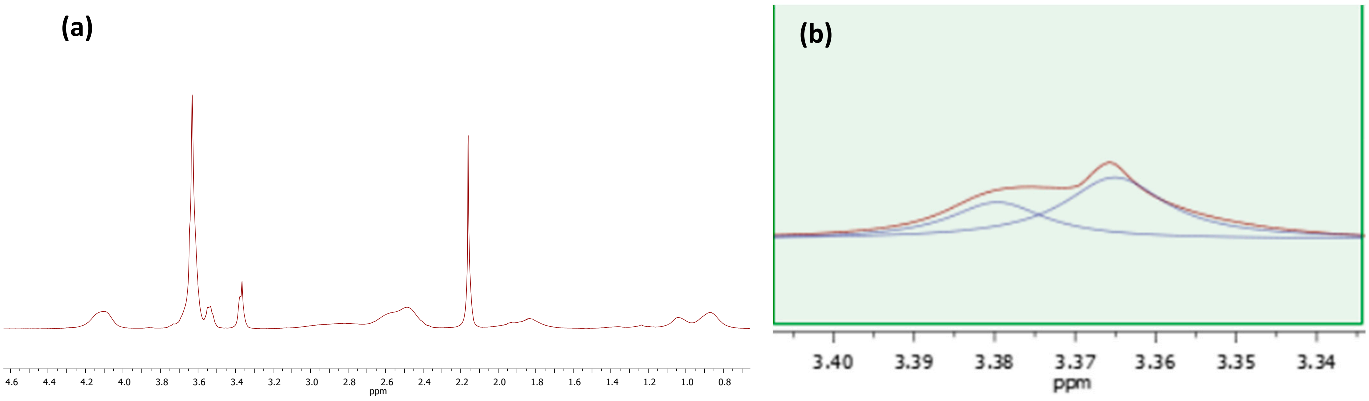

Figure S1. (a) ^{1}H-NMR of P-1 terpolymer **(b)** line fitting of methoxy protons peak.

The deconvolution of the broad peak which corresponds to methoxy protons of DEGMA and OEGMA and ranges from 3.33 to 3.40 ppm, results in the formation of two peaks, one for each methoxy group. The peak of DMAEMA which corresponds to 6 protons of the tertiary amino group (2.16 ppm) was selected as reference peak and was also line fitted, providing an area of 42654. All the aforementioned line fits were executed using the MestreNova software.

Table S1. Line fitting data for methoxy protons of DEGMA and OEGMA of terpolymer P-1

Methoxy group	ppm	Height	Area
DEGMA	3.38	186	5771
OEGMA	3.36	312	10222

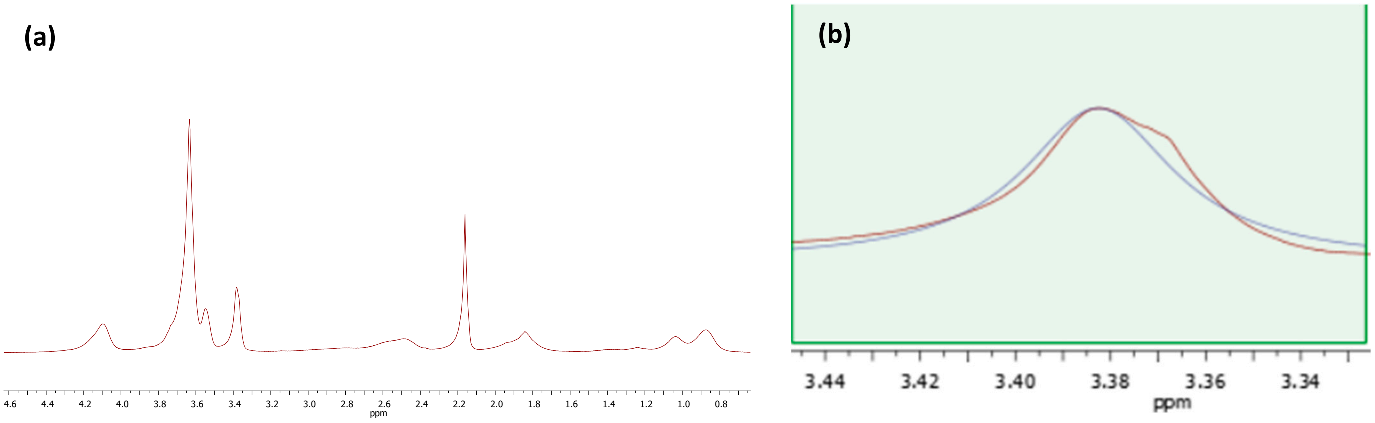

Figure S2. (a) ^{1}H-NMR of P-2 terpolymer **(b)** line fitting of methoxy protons peak.

As presented at Figure S2, the peak of methoxy protons of DEGMA and OEGMA cannot be deconvoluted via line fitting, therefore the calculation of the composition for P-2 terpolymer becomes unfeasible.

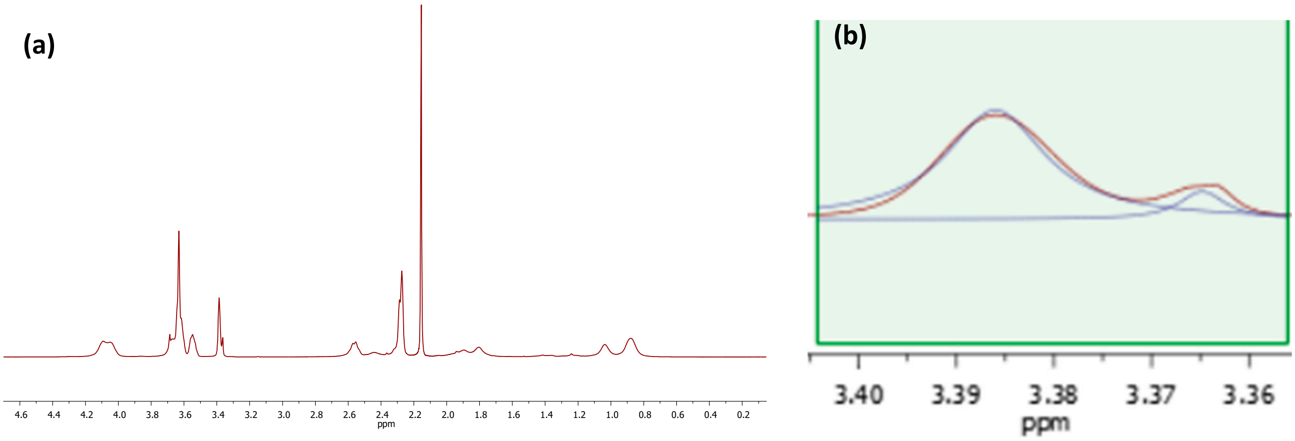

Figure S3. (a) ^{1}H-NMR of P-3 terpolymer **(b)** line fitting of methoxy protons peak.

The deconvolution of methoxy protons broad peak was executed via line fitting as described previously. The peak which corresponds to tertiary amino group protons (2.16 ppm) was selected as the reference peak and via line fitting provided an area of 54520.

Table S2. Line fitting data for methoxy protons of DEGMA and OEGMA of terpolymer P-3

Methoxy group	ppm	Height	Area
DEGMA	3.38	845	22415
OEGMA	3.36	217	2624
